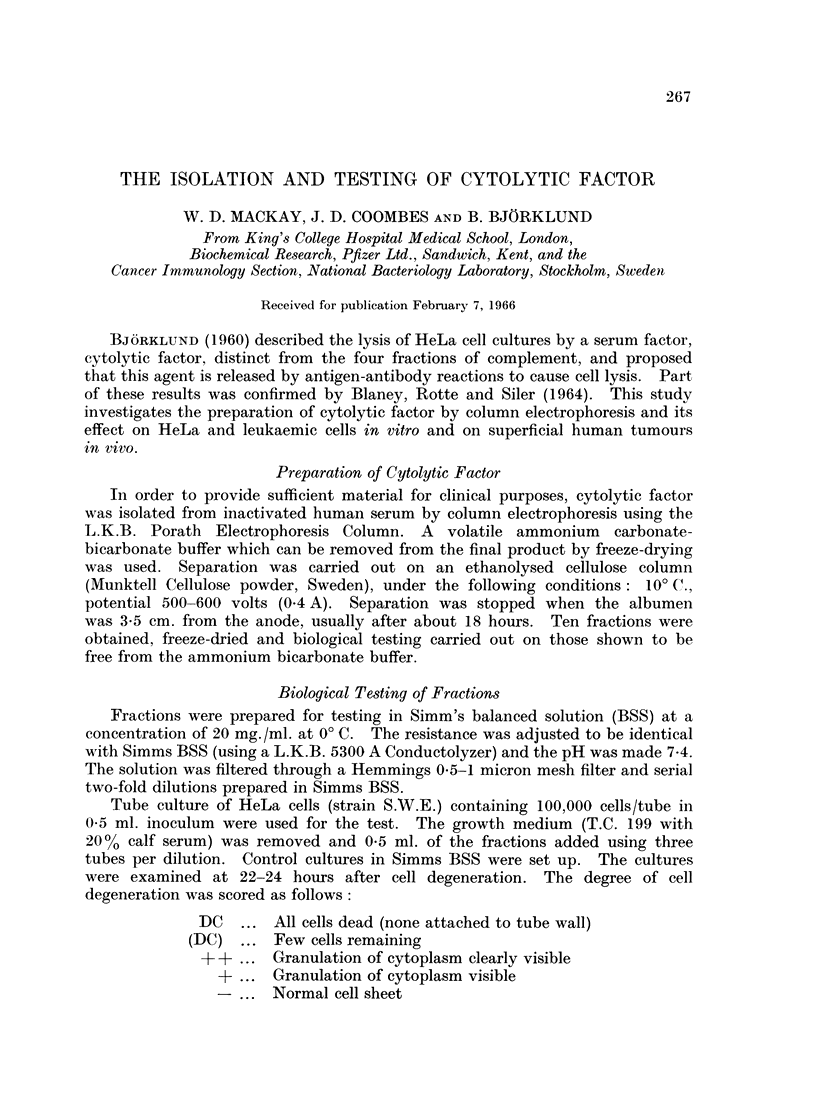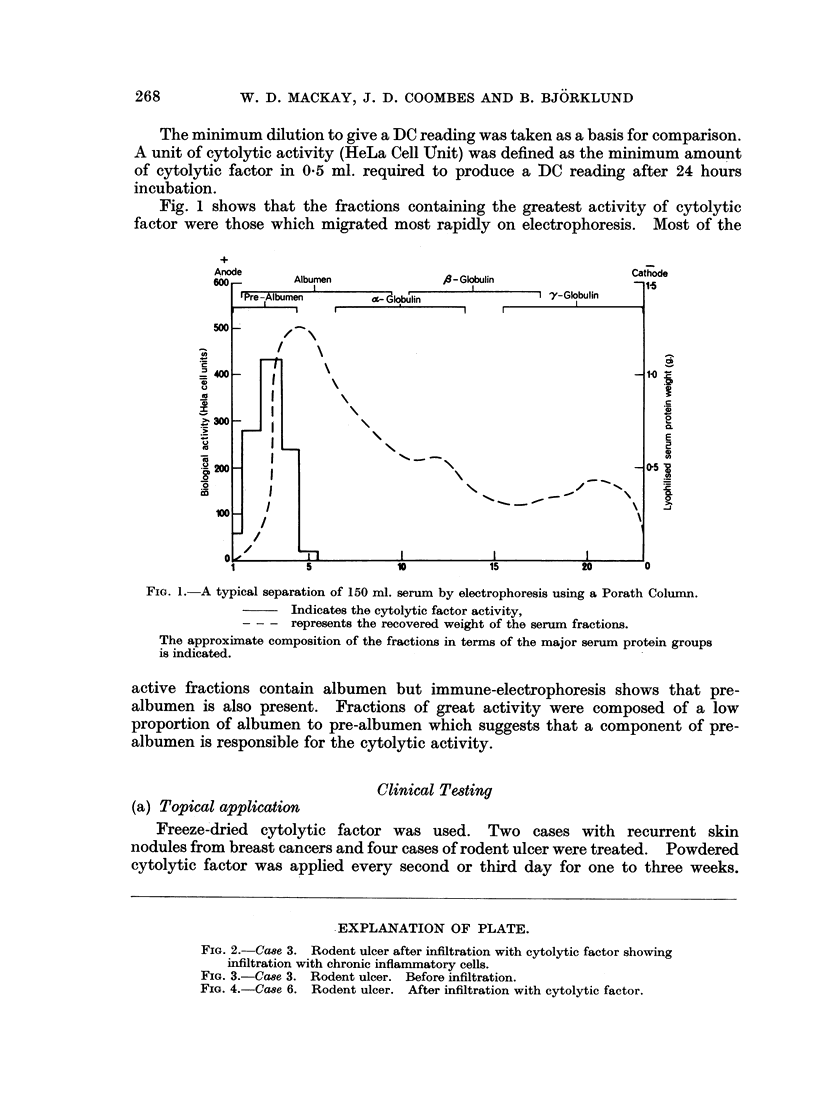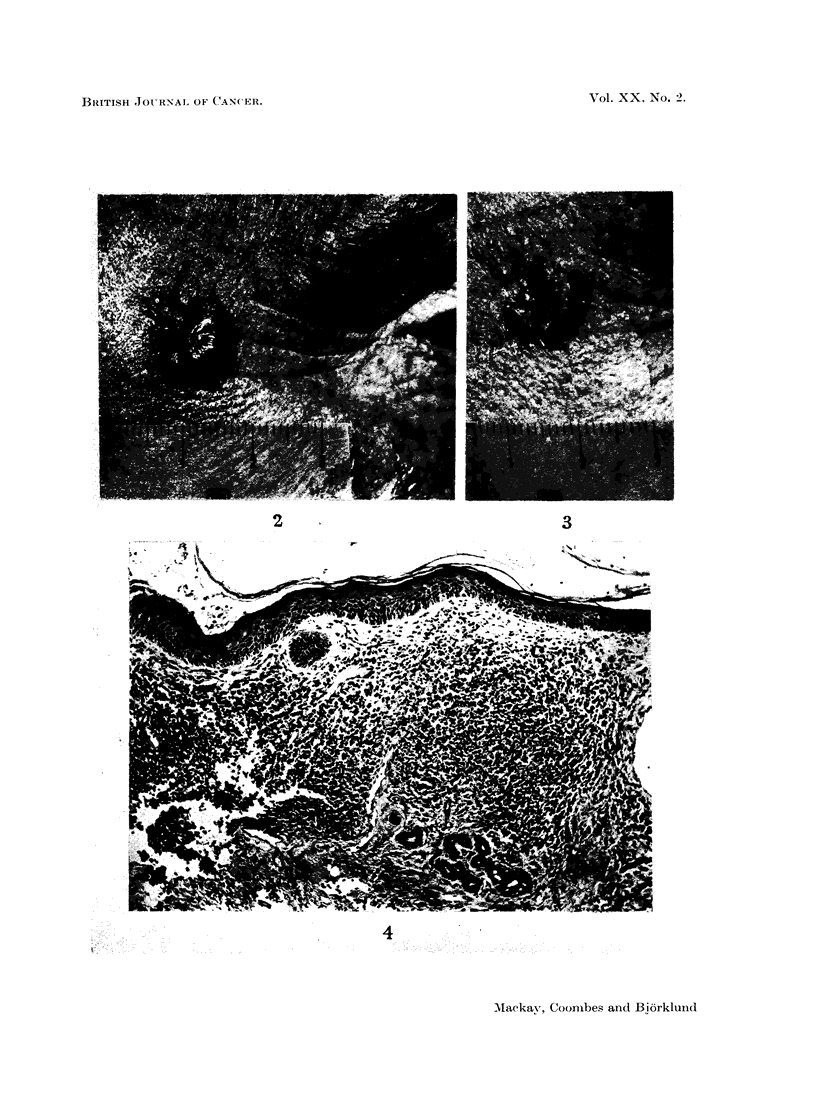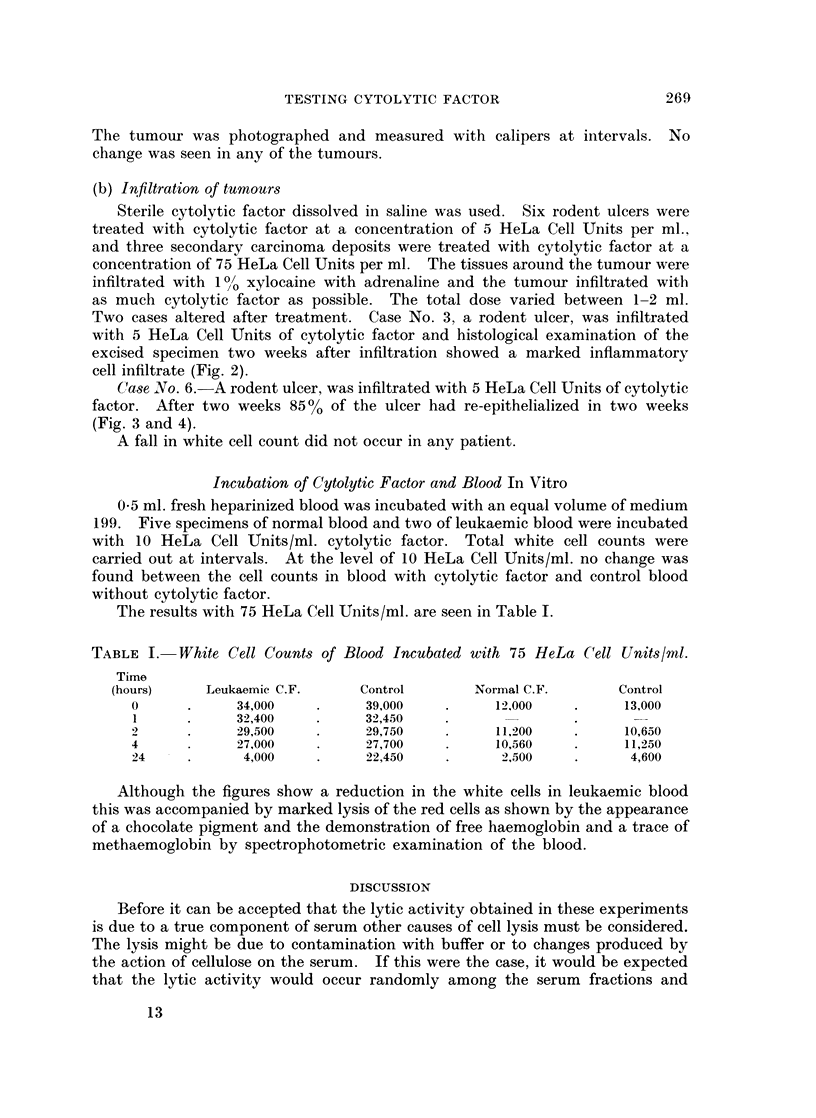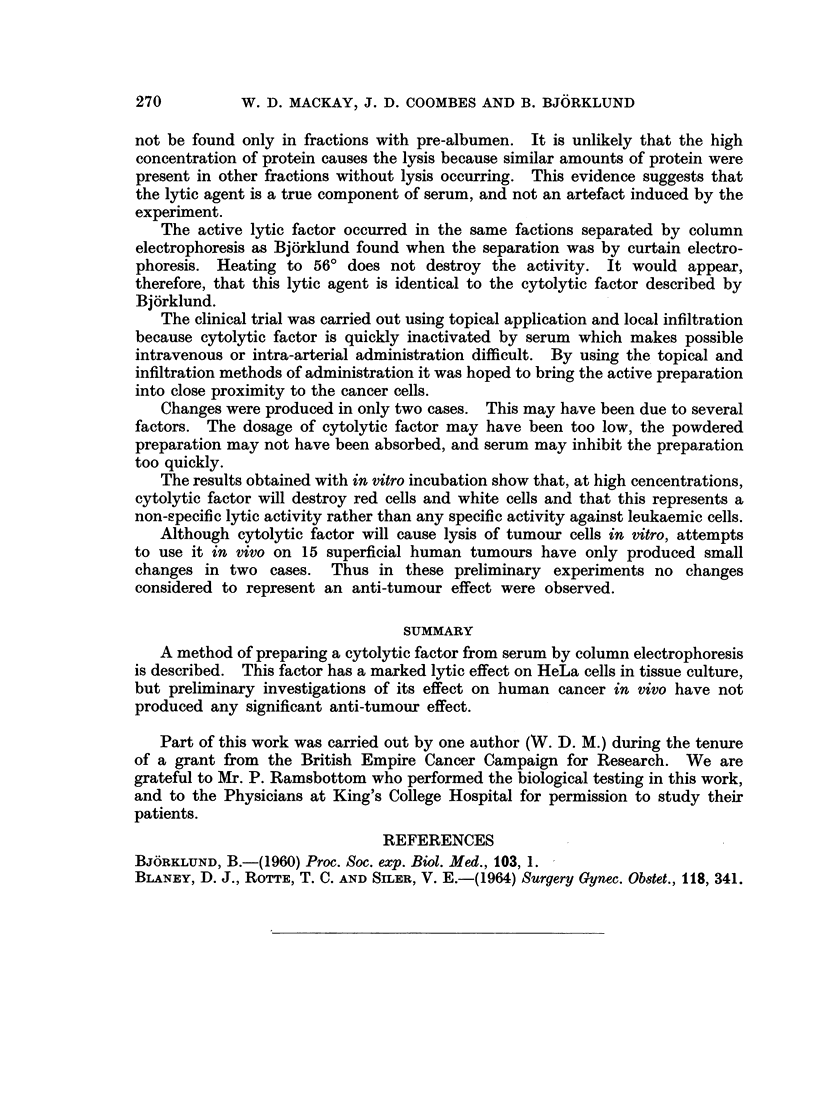# The isolation and testing of cytolytic factor.

**DOI:** 10.1038/bjc.1966.34

**Published:** 1966-06

**Authors:** W. D. Mackay, J. D. Coombes, B. Björklund

## Abstract

**Images:**


### THE ISOLATION AND TESTING OF CYTOLYTIC FACTOR

W. D. MACKAY, J. D. COOMBES AND B. BJÖRKLUND

*From King's College Hospital Medical School, London, Biochemical Research, Pfizer Ltd., Sandwich, Kent, and the Cancer Immunology Section, National Bacteriology Laboratory, Stockholm, Sweden*

Received for publication February 7, 1966

BJÖRKLUND (1960) described the lysis of HeLa cell cultures by a serum factor, cytolytic factor, distinct from the four fractions of complement, and proposed that this agent is released by antigen-antibody reactions to cause cell lysis. Part of these results was confirmed by Blaney, Rotte and Siler (1964). This study investigates the preparation of cytolytic factor by column electrophoresis and its effect on HeLa and leukaemic cells *in vitro* and on superficial human tumours *in vivo*.

### *Preparation of Cytolytic Factor*

In order to provide sufficient material for clinical purposes, cytolytic factor was isolated from inactivated human serum by column electrophoresis using the L.K.B. Porath Electrophoresis Column. A volatile ammonium carbonate-bicarbonate buffer which can be removed from the final product by freeze-drying was used. Separation was carried out on an ethanolysed cellulose column (Munktell Cellulose powder, Sweden), under the following conditions: 10° C., potential 500–600 volts (0·4 A). Separation was stopped when the albumen was 3·5 cm. from the anode, usually after about 18 hours. Ten fractions were obtained, freeze-dried and biological testing carried out on those shown to be free from the ammonium bicarbonate buffer.

### *Biological Testing of Fractions*

Fractions were prepared for testing in Simm's balanced solution (BSS) at a concentration of 20 mg./ml. at 0° C. The resistance was adjusted to be identical with Simms BSS (using a L.K.B. 5300 A Conductolyzer) and the pH was made 7·4. The solution was filtered through a Hemmings 0·5–1 micron mesh filter and serial two-fold dilutions prepared in Simms BSS.

Tube culture of HeLa cells (strain S.W.E.) containing 100,000 cells/tube in 0·5 ml. inoculum were used for the test. The growth medium (T.C. 199 with 20% calf serum) was removed and 0·5 ml. of the fractions added using three tubes per dilution. Control cultures in Simms BSS were set up. The cultures were examined at 22–24 hours after cell degeneration. The degree of cell degeneration was scored as follows:

| | | |
|---|---|---|
| DC | ... | All cells dead (none attached to tube wall) |
| (DC) | ... | Few cells remaining |
| ++ | ... | Granulation of cytoplasm clearly visible |
| + | ... | Granulation of cytoplasm visible |
| — | ... | Normal cell sheet |

The minimum dilution to give a DC reading was taken as a basis for comparison. A unit of cytolytic activity (HeLa Cell Unit) was defined as the minimum amount of cytolytic factor in 0·5 ml. required to produce a DC reading after 24 hours incubation.

Fig. 1 shows that the fractions containing the greatest activity of cytolytic factor were those which migrated most rapidly on electrophoresis. Most of the

Fig. 1.—A typical separation of 150 ml. serum by electrophoresis using a Porath Column.
——— Indicates the cytolytic factor activity,
– – – represents the recovered weight of the serum fractions.
The approximate composition of the fractions in terms of the major serum protein groups is indicated.

active fractions contain albumen but immune-electrophoresis shows that pre-albumen is also present. Fractions of great activity were composed of a low proportion of albumen to pre-albumen which suggests that a component of pre-albumen is responsible for the cytolytic activity.

### *Clinical Testing*

### (a) *Topical application*

Freeze-dried cytolytic factor was used. Two cases with recurrent skin nodules from breast cancers and four cases of rodent ulcer were treated. Powdered cytolytic factor was applied every second or third day for one to three weeks.

---

EXPLANATION OF PLATE.

Fig. 2.—*Case* 3. Rodent ulcer after infiltration with cytolytic factor showing infiltration with chronic inflammatory cells.
Fig. 3.—*Case* 3. Rodent ulcer. Before infiltration.
Fig. 4.—*Case* 6. Rodent ulcer. After infiltration with cytolytic factor.

2

3

4

The tumour was photographed and measured with calipers at intervals. No change was seen in any of the tumours.

(b) *Infiltration of tumours*

Sterile cytolytic factor dissolved in saline was used. Six rodent ulcers were treated with cytolytic factor at a concentration of 5 HeLa Cell Units per ml., and three secondary carcinoma deposits were treated with cytolytic factor at a concentration of 75 HeLa Cell Units per ml. The tissues around the tumour were infiltrated with 1% xylocaine with adrenaline and the tumour infiltrated with as much cytolytic factor as possible. The total dose varied between 1–2 ml. Two cases altered after treatment. Case No. 3, a rodent ulcer, was infiltrated with 5 HeLa Cell Units of cytolytic factor and histological examination of the excised specimen two weeks after infiltration showed a marked inflammatory cell infiltrate (Fig. 2).

*Case No.* 6.—A rodent ulcer, was infiltrated with 5 HeLa Cell Units of cytolytic factor. After two weeks 85% of the ulcer had re-epithelialized in two weeks (Fig. 3 and 4).

A fall in white cell count did not occur in any patient.

### *Incubation of Cytolytic Factor and Blood* In Vitro

0·5 ml. fresh heparinized blood was incubated with an equal volume of medium 199. Five specimens of normal blood and two of leukaemic blood were incubated with 10 HeLa Cell Units/ml. cytolytic factor. Total white cell counts were carried out at intervals. At the level of 10 HeLa Cell Units/ml. no change was found between the cell counts in blood with cytolytic factor and control blood without cytolytic factor.

The results with 75 HeLa Cell Units/ml. are seen in Table I.

TABLE I.—*White Cell Counts of Blood Incubated with 75 HeLa Cell Units/ml.*

| Time (hours) | Leukaemic C.F. | Control | Normal C.F. | Control |
|---|---|---|---|---|
| 0 | 34,000 | 39,000 | 12,000 | 13,000 |
| 1 | 32,400 | 32,450 | — | — |
| 2 | 29,500 | 29,750 | 11,200 | 10,650 |
| 4 | 27,000 | 27,700 | 10,560 | 11,250 |
| 24 | 4,000 | 22,450 | 2,500 | 4,600 |

Although the figures show a reduction in the white cells in leukaemic blood this was accompanied by marked lysis of the red cells as shown by the appearance of a chocolate pigment and the demonstration of free haemoglobin and a trace of methaemoglobin by spectrophotometric examination of the blood.

### DISCUSSION

Before it can be accepted that the lytic activity obtained in these experiments is due to a true component of serum other causes of cell lysis must be considered. The lysis might be due to contamination with buffer or to changes produced by the action of cellulose on the serum. If this were the case, it would be expected that the lytic activity would occur randomly among the serum fractions and not be found only in fractions with pre-albumen. It is unlikely that the high concentration of protein causes the lysis because similar amounts of protein were present in other fractions without lysis occurring. This evidence suggests that the lytic agent is a true component of serum, and not an artefact induced by the experiment.

The active lytic factor occurred in the same factions separated by column electrophoresis as Björklund found when the separation was by curtain electrophoresis. Heating to 56° does not destroy the activity. It would appear, therefore, that this lytic agent is identical to the cytolytic factor described by Björklund.

The clinical trial was carried out using topical application and local infiltration because cytolytic factor is quickly inactivated by serum which makes possible intravenous or intra-arterial administration difficult. By using the topical and infiltration methods of administration it was hoped to bring the active preparation into close proximity to the cancer cells.

Changes were produced in only two cases. This may have been due to several factors. The dosage of cytolytic factor may have been too low, the powdered preparation may not have been absorbed, and serum may inhibit the preparation too quickly.

The results obtained with *in vitro* incubation show that, at high cencentrations, cytolytic factor will destroy red cells and white cells and that this represents a non-specific lytic activity rather than any specific activity against leukaemic cells.

Although cytolytic factor will cause lysis of tumour cells *in vitro*, attempts to use it *in vivo* on 15 superficial human tumours have only produced small changes in two cases. Thus in these preliminary experiments no changes considered to represent an anti-tumour effect were observed.

### SUMMARY

A method of preparing a cytolytic factor from serum by column electrophoresis is described. This factor has a marked lytic effect on HeLa cells in tissue culture, but preliminary investigations of its effect on human cancer *in vivo* have not produced any significant anti-tumour effect.

Part of this work was carried out by one author (W. D. M.) during the tenure of a grant from the British Empire Cancer Campaign for Research. We are grateful to Mr. P. Ramsbottom who performed the biological testing in this work, and to the Physicians at King's College Hospital for permission to study their patients.

### REFERENCES

BJÖRKLUND, B.—(1960) *Proc. Soc. exp. Biol. Med.*, **103**, 1.
BLANEY, D. J., ROTTE, T. C. AND SILER, V. E.—(1964) *Surgery Gynec. Obstet.*, **118**, 341.